# Next-Generation Mitogenomics: A Comparison of Approaches Applied to Caecilian Amphibian Phylogeny

**DOI:** 10.1371/journal.pone.0156757

**Published:** 2016-06-09

**Authors:** Simon T. Maddock, Andrew G. Briscoe, Mark Wilkinson, Andrea Waeschenbach, Diego San Mauro, Julia J. Day, D. Tim J. Littlewood, Peter G. Foster, Ronald A. Nussbaum, David J. Gower

**Affiliations:** 1 Department of Life Sciences, Natural History Museum, London, SW7 5BD, United Kingdom; 2 Department of Genetics, Evolution and Environment, University College London, London, WC1E 6BT, United Kingdom; 3 Department of Animal Management, Reaseheath College, Nantwich, CW5 6DF, United Kingdom; 4 Department of Zoology and Physical Anthropology, Complutense University of Madrid, 28040, Madrid, Spain; 5 Museum of Zoology, University of Michigan, Ann Arbor, MI, 48109–1079, United States of America; 6 Department of Ecology and Evolutionary Biology, University of Michigan, Ann Arbor, MI, 48109–1079, United States of America; Sichuan University, CHINA

## Abstract

Mitochondrial genome (mitogenome) sequences are being generated with increasing speed due to the advances of next-generation sequencing (NGS) technology and associated analytical tools. However, detailed comparisons to explore the utility of alternative NGS approaches applied to the same taxa have not been undertaken. We compared a ‘traditional’ Sanger sequencing method with two NGS approaches (shotgun sequencing and non-indexed, multiplex amplicon sequencing) on four different sequencing platforms (Illumina’s HiSeq and MiSeq, Roche’s 454 GS FLX, and Life Technologies’ Ion Torrent) to produce seven (near-) complete mitogenomes from six species that form a small radiation of caecilian amphibians from the Seychelles. The fastest, most accurate method of obtaining mitogenome sequences that we tested was direct sequencing of genomic DNA (shotgun sequencing) using the MiSeq platform. Bayesian inference and maximum likelihood analyses using seven different partitioning strategies were unable to resolve compellingly all phylogenetic relationships among the Seychelles caecilian species, indicating the need for additional data in this case.

## Introduction

Technological advancement and decreasing costs have increased the use of high-throughput sequencing platforms in evolutionary biology [1]. Several recent studies have generated mitogenomic data sets for phylogenetics using next-generation sequencing (NGS) [2–5], with either long-range PCRs [4] or shotgun sequencing [2] and using a variety of sequencing platforms. Some studies have examined sequencing platform performance [6,7] but detailed comparisons and evaluations of different NGS approaches for mitogenomic phylogenetics of the same set of taxa have not been carried out.

Here we present a comparison of four different NGS approaches for generating (near-) complete mitogenome DNA sequences. Two primary methods were employed: 1) multiplex sequencing of pooled, non-indexed long-range PCR products from a multitude of taxa [5] using three different platforms: HiSeq (Illumina), 454 GS FLX (Roche), and Ion Torrent (Life Technologies), and 2) individually indexed shotgun sequencing of genomic DNA [8] using the MiSeq platform (Illumina).

We explored the efficacy of various approaches for generating complete mitogenome DNA sequences for a clade of caecilian amphibians (Gymnophiona) endemic to the Seychelles. Mitogenomic data have played an especially important role in recent advances in the understanding of caecilian phylogeny, systematics, and evolution [9–14]. Caecilian mitogenomes have also provided the best evidence for tandem duplication and random loss as a mechanism of mitochondrial gene order rearrangements [15], and have been used in studies of experimental design in phylogenetics [9,10]. However, mitogenomes have only partly been applied, thus far, to the ongoing problem of the relationships among the Seychelles caecilians. The Seychelles caecilians comprise a radiation [16–21] of six nominal species in three genera (*Grandisonia alternans*, *G*. *larvata*, *G*. *sechellensis*, *Hypogeophis brevis*, *H*. *rostratus*, *Praslinia cooperi*) within the family Indotyphlidae (following the classification of Wilkinson et al. [22]). Prior to 2009, analyses of small fragments of mtDNA sequence data had reached no consensus beyond that the radiation is monophyletic and that the monotypic *Praslinia* is sister to all other Seychelles species [16,17,21,23,24]. More recently, complete [11] or near-complete [14] mitogenomes have been generated for four of the Seychelles species, but this limited taxon sampling precluded comprehensive phylogenetic insights. Resolution of the phylogenetic relationships among the Seychelles caecilians would be beneficial in helping to stabilise their genus-level taxonomy [22], and in providing a platform for more detailed analysis of the evolution of reproductive traits within indotyphlids, which likely includes the re-evolution of a larval stage [11].

## Methods

### Taxon sampling and DNA extraction

Six Sanger-sequenced complete or near-complete mitogenome sequences had been previously generated for four of the six nominal species of Seychelles caecilians [11,14] (see Table 1). These mitogenomes were generated using multiple primer pairs designed to amplify 14 [11] or 13 [14] overlapping fragments. We attempted to generate sequences of a further eight mitogenomes for five Seychelles species using four NGS approaches. Samples were obtained from the frozen tissue collection of the University of Michigan Museum of Zoology, USA (voucher specimen codes with the prefix UMMZ; some incompletely accessioned material with RAN prefix). For three individuals (*G*. *alternans* UMMZ240022, *G*. *larvata* UMMZ240023, *H*. *brevis* UMMZ192977), mitogenomic data were generated using more than one method. Our sampling (Table 1) included the two Seychelles caecilian species (*G*. *alternans*, *H*. *brevis*) not previously sampled for mitogenomes and whose sister taxa are not resolved [16,21,23,24].

**Table 1:** Voucher specimen (codes refer to vouchers: RAN = RAN’s field numbers; UMMZ = University of Michigan Museum of Zoology, Ann Arbor; MVZ = Museum of Vertebrate Zoology, Berkeley) and associated mitogenome sequence information for the six nominal species of Seychelles caecilian (species of *Grandisonia*, *Hypogeophis*, *Praslinia*). GenBank codes in bold were published previously. bp = base pairs; Av. Cov. = average read coverage across mitogenome. * = genome sequence not fully complete; (1) = voucher incorrectly identified as *G*. *alternans* by Zhang & Wake (2009: see San Mauro et al. 2014). ^*#*^ = specimen that was excluded from phylogenetic analysis due to the mitogenome sequence being substantially incomplete.

Species	Voucher	GenBank code	Published	bp—total	MiSeq (Av. Cov.)	bp—HiSeq	bp—454	bp—Ion Torrent	GC %
*G*. *alternans*	RAN31062	KU753811	This study	16,065	20.7	-	-	-	38.5
*G*. *alternans*	UMMZ240022	KU974367	This study	14,827	-	14,343	14,019	10,743	36.1
*G*. *alternans*	UMMZ192945	KU753815	This study	14,836	7.4	-	-	-	36.6
*G*. *larvata*^*#*^	UMMZ240023	KU753812	This study			6,471	5,846	5,406	
*G*. *larvata*	RAN31203	KU753813	This study	15,388	7.1	-	-	-	33.6
*G*. *larvata* (1)	MVZ258026	GQ244470*	Zhang & Wake, 2009	15,209	-	-	-	-	34.8
*G*. *sechellensis*	UMMZ193076	KU753816	This study	16,071	20.7	-	-	-	36.2
*G*. *sechellensis*	UMMZ240024	**KF540152**	San Mauro et al., 2014	16,094	-	-	-	-	36.3
*H*. *brevis*	UMMZ192977	KU753817	This study	16,107	39	15,540	15,578	9,593	35.9
*H*. *rostratus*	RAN31219	KU753814	This study	10,782	2.5	-	-	-	26.3
*H*. *rostratus*	MVZ258025	**GQ244472**	Zhang & Wake, 2009	16,151	-	-	-	-	35.8
*H*. *rostratus*	UMMZ240025	**KF540154**	San Mauro et al., 2014	16,170	-	-	-	-	35.4
*P*. *cooperi*	UMMZ192933	GQ244475*	Zhang & Wake, 2009	15,218	-	-	-	-	38.4
*P*. *cooperi*	UMMZ192934	**KF540162**	San Mauro et al., 2014	16,192	-	-	-	-	38

Liver and/or muscle samples of Seychelles caecilians were obtained during fieldwork between 1988 and 1991. Animals were collected by digging with hoes and by turning logs and rocks. Fieldwork was carried out with the permission of Seychelles Bureau of Standards; permission for the collection of specimens and issuing of export permits was provided by Seychelles Department of Environment. No ethical approval was required for this work because no experimentation was carried out, although, the University of Michigan Animal Care Unit (UCUCA) approved all methods. At the time of collection none of the species used in this study had been assessed by the IUCN Red List of Threatened Species. Specimens were anaesthetized with chlorotone and fixed in 5% formalin before being stored in 70% EtOH at the University of Michigan Museum of Zoology, Ann Arbor, USA (UMMZ); fresh tissue samples from sacrificed animals were frozen at -80°C. Genomic DNA was extracted using the DNeasy Blood and Tissue Kit (QIAGEN), following manufacturer’s guidelines with the exception of the final suspension solution, which was modified to 2x100μl of buffer AE (the first elution was used in all subsequent analyses).

### gDNA shotgun sequencing using the MiSeq (Illumina) platform

Next-generation sequencing libraries for six individual samples (two *G*. *alternans*; one of each of *G*. *larvata*, *G*. *sechellensis*, *H*. *brevis* and *H*. *rostratus*), destined for shotgun sequencing, were prepared for Illumina MiSeq sequencing using a standard Illumina Nextera DNA kit. The primary aim of this sequencing run was to develop anonymous nuclear markers [25]. Paired-end reads (≤251bp long) were sequenced using a 500 cycle v.2 reagent kit on a single MiSeq flow cell. Each sample was indexed so that all sequences could be individually identified.

The paired-end MiSeq data were combined for each sample and subsequently cleaned with the Trim Ends function in Geneious v.6.1.4 (Biomatters) using default settings. FASTQ files containing the paired-end data were run through the MITObim pipeline (100 iterations;—quick option) using the six previously published Seychelles caecilian mitogenomes [11,14] as a reference. MitoBim was chosen because of its reported superiority over other mapping tools [26]. However, initial runs for each sample yielded reconstructed mitogenomes with approximately 500 base pairs (bp) missing from the end of the assembly. To combat this, 1,000bp of the linear reference mitogenomes were moved from the end to the start of the alignment and analyses were rerun. Both runs for each specimen were then compared, aligned against each other, trimmed, and a consensus sequence was produced in Geneious.

### Multiplex amplicon sequencing using HiSeq (Illumina), 454 GS FLX (Roche) and Ion Torrent (Life Technologies) platforms

The complete mitogenomes of *G*. *alternans* (UMMZ240022) and *H*. *brevis* (UMMZ192977) along with the partial mitogenome (6,471 bp) of *G*. *larvata* (UMMZ240023) were sequenced in parallel with 475 non-indexed long-range mitogenomic PCR amplicons from 270 other animal taxa (including some caecilians), as part of a larger project.

Long-range PCRs were carried out in 50 μl reaction volumes using the Expand 20kb^PLUS^ PCR System (Roche) using 4 μl of gDNA following manufacturers’ recommendations. The mitogenomes were amplified in two overlapping fragments, ~6.4kb and ~10.7kb, using the primer pairs Amp-12S.F (5’-AAGAAATGGGCTACATTTTCT-3’) + Amp-P3.R (5’-GCTTCTCARATAATAAATATYAT-3’) and Amp-P4.F (5’-GGMTTTATTCACTGATTYCC-3’) + Amp-12S.R (5’-TCGATTATAGAACAGGCTCCTCT-3’) [12], respectively, however, the ~10.7kb fragment failed to amplify for *G*. *larvata* (UMMZ240023). Because of the degeneracy of primers Amp-P3.R and Amp-P4.F, 4 μl of 10 μM primer were added to each reaction, whereas only 2 μl were used for Amp-12S.F and Amp-12S.R. The PCR cycling profile for Amp-12S.F + Amp-P3.R was as follows: initial denaturation for 2 min at 92°C, followed by 10 cycles of 15 s at 92°C, 30 s at 45°C, 4 min at 68°C, followed by 30 further cycles in which the extension time was lengthened by 10 s per cycle, and terminated with a final extension of 10 min at 68°C. The PCR cycling profile for Amp-P4.F + Amp-12S.R was as follows: initial denaturation for 2 min at 92°C, followed by 10 cycles of 15 s at 92°C, 30 s at 48°C, 9 min at 68°C, followed by 30 further cycles in which the extension time was lengthened by 10 s per cycle, and terminated with a final extension of 10 min at 68°C. PCR products were purified using QIAquick PCR Purification Kit (QIAGEN) and quantified using a NanoDrop spectrophotometer (Thermo Scientific). An equimolar solution of all 475 amplicons was prepared for NGS sequencing using the Illumina HiSeq, Roche 454 and Ion Torrent platforms on a single lane or flow cell. Short fragments of mtDNA (*12S* and *16S* rRNA, *cox1*, *cytb*) that had been Sanger sequenced for each species [16,17] were used as seeds for read assembly (see below) and to provide amplicon identity.

### Initial reduction of Illumina HiSeq dataset

Because the Illumina HiSeq platform produces a vast amount of data (and because the samples were not individually indexed), the full dataset, which consisted of 270 individual animals, was subjected to an initial reduction to facilitate mitogenome reconstruction for Seychelles caecilians. Three previously published (Sanger-sequenced) Seychelles caecilian mitogenomes (*G*. *sechellensis*, *H*. *rostratus*, *P*. *cooperi*; GenBank accessions KF540152, KF540152, KF540162 respectively) plus one of a proximate outgroup (the Indian indotyphlid *Indotyphlus maharashtraensis*, GenBank accession KF540157) were aligned using Muscle [27] in Geneious with default settings. The alignment was checked by eye and obvious mistakes corrected manually.

The alignment was then viewed in Geneious with a sliding window in order to partition it into blocks within which the four mitogenomes had similar magnitudes of sequence (dis)similarity. Separate sub-alignments were generated for each of 16 such regions, the sub-alignments ranging in size from 289–2,525bp (each overlapping by at least 50bp with neighbouring alignments to counter potential loss of reads) (Table 2). The maximum sequence divergence (*p*-distance) among the four mitogenomes was calculated from the sliding window for each of the 16 sub-alignments. A consensus sequence was generated for each sub-alignment and used as references for mapping assemblies in order to extract caecilian reads from the raw, non-indexed HiSeq data, using a mismatch threshold of the maximum divergence among the four mitogenome sequences in each sub-alignment, plus an additional 10% allowance, per read. The additional 10% allowance was an arbitrary threshold that intended to ensure that all of the Seychelles caecilian sequence reads were pulled from the raw data. Reference assemblies were carried out in Geneious with the following parameters: single iteration mapping assembly, 15% gaps allowed per read, maximum gap size 50, word length 14, index word length 12, maximum ambiguity 4 (allowing 1 ambiguous base per read) and the number of mismatches allowed per read as described above. From this point, these initially reduced Illumina HiSeq data were subject to the same treatment as the Roche 454 and Ion Torrent data.

**Table 2:** Size ranges used to partition the Illumina HiSeq dataset into a manageable size based on a sliding window analysis. Position 0 refers to the start of the *trnF(gaa)* tRNA gene.

Position in alignment (bp)	Maximum sequence divergence (%)
0–1,076	22
976–2,855	21
2,779–4,036	20
3,823–5,073	24
4,973–5,461	20
5,361–7,146	18
7,043–7,837	19
7,787–8,076	27
8,004–8,753	24
8,653–9,604	19
9,537–10,328	26
10,228–11,789	24
11,689–14,214	24
14,114–15,427	21
15,327–16,440	35
16,087–354	30

### Mitogenome reconstruction from Roche 454, Ion Torrent and distilled Illumina HiSeq data

Each of the three amplicon data sets were assembled in Geneious using the “map to reference” function with the four Sanger sequenced seeds used as references (see above). The assemblies were performed for 100 iterations with the following settings: 3% mismatches per read, maximum gap size of 15, maximum overlap identity of 80%, maximum ambiguity 1, and multiple best matches mapped randomly.

In order to locate relevant reads that might have been discounted in assemblies generated from the starting Sanger seeds (especially for the lower-coverage Ion Torrent data), we used mitogenomes of the same species (previously published Sanger-sequenced data available in every case, except MiSeq indexed for *G*. *alternans*) as references for the “map to reference” option in Geneious, and used the same settings described in the previous paragraph, except for a maximum mismatches per read of 1% and maximum ambiguity of 2. These setting modifications were applied in order to accommodate intraspecific variation.

### Mitogenome annotation and alignment

Alignments of mitogenomic data generated from different platforms for single specimens (available for three specimens: *G*. *alternans* UMMZ240022, *G*. *larvata* UMMZ240023, *H*. *brevis* UMMZ192977) were created using the *de novo* assembler in Geneious v.6.1.6. No major errors were detected by eye and a consensus sequence for each specimen was accepted as the final sequence for further annotation and analysis.

The six previously published (Sanger-sequenced) Seychelles caecilian mitogenome sequences were aligned using Muscle in Geneious with default settings; any obvious misalignments within tRNA genes were corrected manually. The newly generated sequences were then added and aligned using Geneious Consensus Align, maintaining existing gaps, with 70% similarity, gap open penalty of 12, and a gap extension penalty of 3. All novel mitogenomes were compared with those previously published and Sanger seeds (see above) to increase the likelihood of correct reconstruction of the data. When checked, only the new tRNA gene sequences had (very small) obvious mistakes that were attributable to misalignment rather than sequencing or reconstruction error, and these were sought and removed using GBlocks [28] using the “with half” setting.

The initial annotation of the newly reconstructed mitogenomes was carried out using MITOS [29], BLASTn [30], and by alignment against the six previously published Seychelles caecilian mitogenomes [11,14]. The final annotation was undertaken manually in Geneious. When annotating protein-coding genes, information was incorporated from codon position determined using MEGA v.6.06 [31]. GenBank accession numbers for newly generated sequences can be found in Table 1.

### Phylogenetic analysis

Following San Mauro *et al*. [9–11], the regulatory, non-coding L-strand replication and control regions were removed from the alignment. Best-fit models of nucleotide substitution and data-partition schemes were determined using PartitionFinder v.1.1.1 [32] for five datasets, comprising all or subsets of the concatenated first, second and third codon positions of protein coding genes, concatenated rRNA genes, and concatenated tRNA genes (total of 15,399 aligned bp excluding ambiguously aligned sites which were removed).

Phylogenetic trees were inferred using Bayesian inference (BI) and maximum likelihood (ML) algorithms implemented in the programs MrBayes v.3.2.2 [33] and RaxML v.8.0.24 [34], respectively and run through the CIPRES Science Gateway server [35]. For BI, the five datasets described in the previous paragraph were each subjected to two independent analyses. Optimal partitioning strategies and best-fit models as determined by PartitionFinder are given in Table 3. The BI analysis was run for 10^7^generations and sampled every 10,000 generations with one cold and three heated chains, with the first 10% of trees discarded as burn-in. Chains were checked for convergence using Tracer v1.5 [36] by assessing ESS scores and by visualization of mixing on the trace. For the ML analyses the Blackbox option was employed using default options [37].

**Table 3:** Summary information for mitogenome data partitions and their best-fit models. All data are for nucleotides, except “Amino Acid”. CS = number of constant sites, PI = number of parsimony informative sites, CP1, 2, 3 = protein-coding codon position 1, 2 and 3.

Data	Sites	CS	PI	Partitions and models
All	15,399	10,534	4,241	CP1, rRNA, tRNA (GTR+G); CP2, CP3 (GTR+I+G)
Protein Coding genes	11,272	7,224	3,425	CP1, CP2, CP3 (GTR+I+G)
tRNAs	1,600	1,241	300	GTR+I+G
rRNAs	2,527	1,927	516	GTR+I+G
Amino Acid	3,746	2,996	617	

Potential saturation of third-codon positions was assessed using the method described by Xia et al. [38] in DAMBE v.5 [39]; PAUP* v.4.0a136 [40] was used to test for base composition heterogeneity and, where found, bootstrap (1000 replicates) LogDet/paralinear [41,42] distance analyses using the minimum evolution algorithm with default parameters were also carried out.

BI of the amino acid dataset was conducted using PhyloBayes [43]. PhyloBayes implements the CAT model [44] which allows for site-specific rates of mutation and is often considered a more realistic model of amino acid evolution, and being well suited to larger multigene alignments. Two independent runs were carried out implementing the CAT and the GTRCAT models. MCMC chains ran for at least 40,000 cycles and convergence was assessed when the “maxdiff” parameter was < 0.1. Approximately 25% of trees were discarded as burn-in and remaining trees were sampled every 100 generations.

Phylogenies were rooted with *Praslinia cooperi* based on prior evidence that this taxon is sister to all other Seychelles caecilians. This phylogenetic relationship has been recovered by all published analyses of molecular data [10,11,16,17,21,23,24], except those of Pyron & Wiens [45] and Pyron [46], who recovered *Grandisonia alternans* as the sister group instead. We consider the latter problematic due in part to the extensive outgroups used (MW, unpublished) and disregard them here.

To investigate taxon instability and any impact this might have upon support, we interrogated sets of bootstrap or Bayesian trees with the intersection algorithm described by Wilkinson [47] and implemented in REDCON 3.0 (http://www.nhm.ac.uk/research-curation/research/projects/software/), which returns a comprehensive summary of the support (frequency of occurrence) for all full and partial (i.e., not including all taxa) splits in a set of trees. These analyses were performed on subsamples of 1,000 trees drawn randomly from the full samples of Bayesian trees.

## Results

### Next-generation mitochondrial genome sequences

Seven near-complete mitogenomes were reconstructed with varying degrees of quality and coverage. All of the trialled methods used in this study provided reasonable coverage of the mitogenomes, apart from the Ion Torrent multiplex approach. The Illumina HiSeq multiplex data produced the greatest coverage, followed by the shotgun-sequenced Illumina MiSeq and Roche 454 data (Table 4).

**Table 4:** Coverage data and total length of mitogenome sequences generated by different platforms. Coverage data for each platform is reported as number of sequence reads used and approximate number of bp in parentheses based on the mean read length (RL). The total lengths of reconstructed mitogenomes are reported under the MtL (mitogenome length in bp) column. Numbers in parentheses within the header row refer to mean RL for each platform.

Species	Sample code	MtL	MiSeq (448 bp)	HiSeq (95 bp)	454 (523 bp)	Ion Torrent (98 bp)
*G*. *alternans*	RAN31062	16,065	6,008 (2,691,584)	-	-	-
*G*. *larvata*	UMMZ240023		-	442,600 (42,047,000)	2,481 (1,297,563)	264 (25,872)
*G*. *larvata*	RAN31203	15,388	562 (251,776)	-	-	-
*H*. *rostratus*	RAN31219	10,782	284 (127,232)	-	-	-
*G*. *alternans*	UMMZ240022	14,827	-	512,609 (48,697,855)	1,178 (616,064)	367 (35,966)
*G*. *sechellensis*	UMMZ193076	16,071	1,655 (741,440)	-	-	-
*G*. *alternans*	UMMZ192945	14,836	583 (261,184)	-	-	-
*H*. *brevis*	UMMZ192977	16,107	3,092 (1,385,216)	670,560 (63,703,200)	2,148 (1,123,404)	375 (36,750)

For the *G*. *larvata* sample sequenced using the multiplex methods (UMMZ 240023), approximately only one third (i.e. 5,787 bp; see Table 1) of the mitogenome was obtained, which represented a single long amplicon. This single amplicon did however have a high coverage of reads for it—the highest of any sample when compared to the length of the final sequence (Table 4).

Of the three multiplex sequencing methods, the Ion Torrent approach was least successful. Considerably fewer reads were obtained and single phantom nucleotides were present (as determined by comparison with data generated using Illumina HiSeq and MiSeq, Roche 454, and Sanger sequencing). The phantom single nucleotides comprised between 0.28 and 0.43% of the total reconstructed sequences (Table 5). Conversely, the mitogenome of *H*. *brevis* (UMMZ192977), reconstructed from Roche 454 multiplex data, contained eight phantom single nucleotide insertions (as judged by comparison with data generated from the Illumina HiSeq and MiSeq, and Ion Torrent platforms used for the same sample), accounting for only 0.05% of the reconstructed sequence (Table 5). All other mitogenome reconstructions that we generated with the multiplex approach (regardless of sequencing platform) and the MiSeq shotgun sequencing approach lacked evidence of phantom nucleotide insertions. The newly generated mitochondrial genome sequences (*H*. *brevis* and *G*. *alternans*) conform to the vertebrate consensus organization [48, 49] in terms of gene content and order.

**Table 5:** Number of single base pairs (bp) that were incorrectly called in the three long-amplicon multiplexed mitogenome sequences, as inferred from consensus reads across the sequencing platform data.

	UMMZ240023 Ion Torrent	UMMZ240022 Ion Torrent	UMMZ192977 Ion Torrent	UMMZ192977 454
A	4	11	6	1
C	1	5	5	3
G		2	2	
T	2	12	10	2
N	8	16	6	2
Insertions added		5		
Total bp	5406	10746	9593	15540

### Mitogenomic phylogeny of Seychelles caecilians

**Fig 1:**
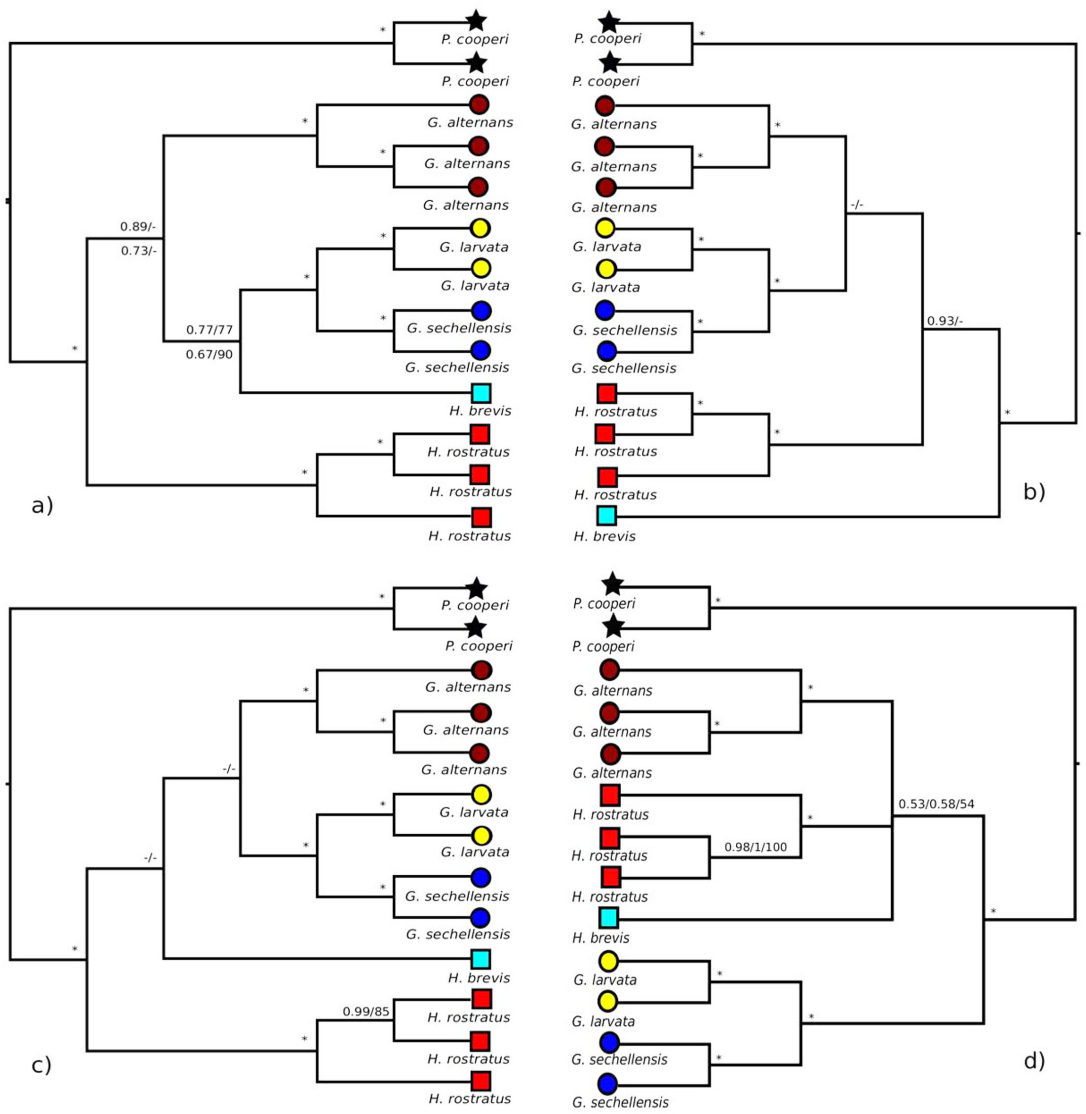
The four phylogenetic tree topologies inferred from the five data sets. (a) for both the complete nucleotide data and the protein-coding nucleotide data (b) rRNA, (c) tRNA (d) amino acids. In (a) numbers above branches are support for the complete nucleotide data and below for the protein-coding nucleotides (BI/ML). In (b) and (c) numbers above branches are for analyses with BI/ML. In (d) values above branches are Bayesian posterior probabilities for the unpartitioned CAT and CATGTR analyses run on PhyloBayes/ and BI/ML support for the gene-partitioned dataset. Maximal support is indicated by a single * and support values below 0.5/50% (BI/ML) are indicated by “-”(or by collapsed branches in the PhyloBayes tree (d)). Symbols at terminals refer to genus: stars = *Praslinia*; squares = *Hypogeophis*; circles = *Grandisonia*. Colours refer to species: black = *P*. *cooperi*; red = *H*. *rostratus*; turquoise = *H*. *brevis*; brown = *G*. *alternans*; yellow = *G*. *larvata*; blue = *G*. *sechellensis*. All trees were rooted with *Praslinia cooperi*. Source trees and branch lengths are deposited online with the Natural History Museum data repository.

**Fig 2:**
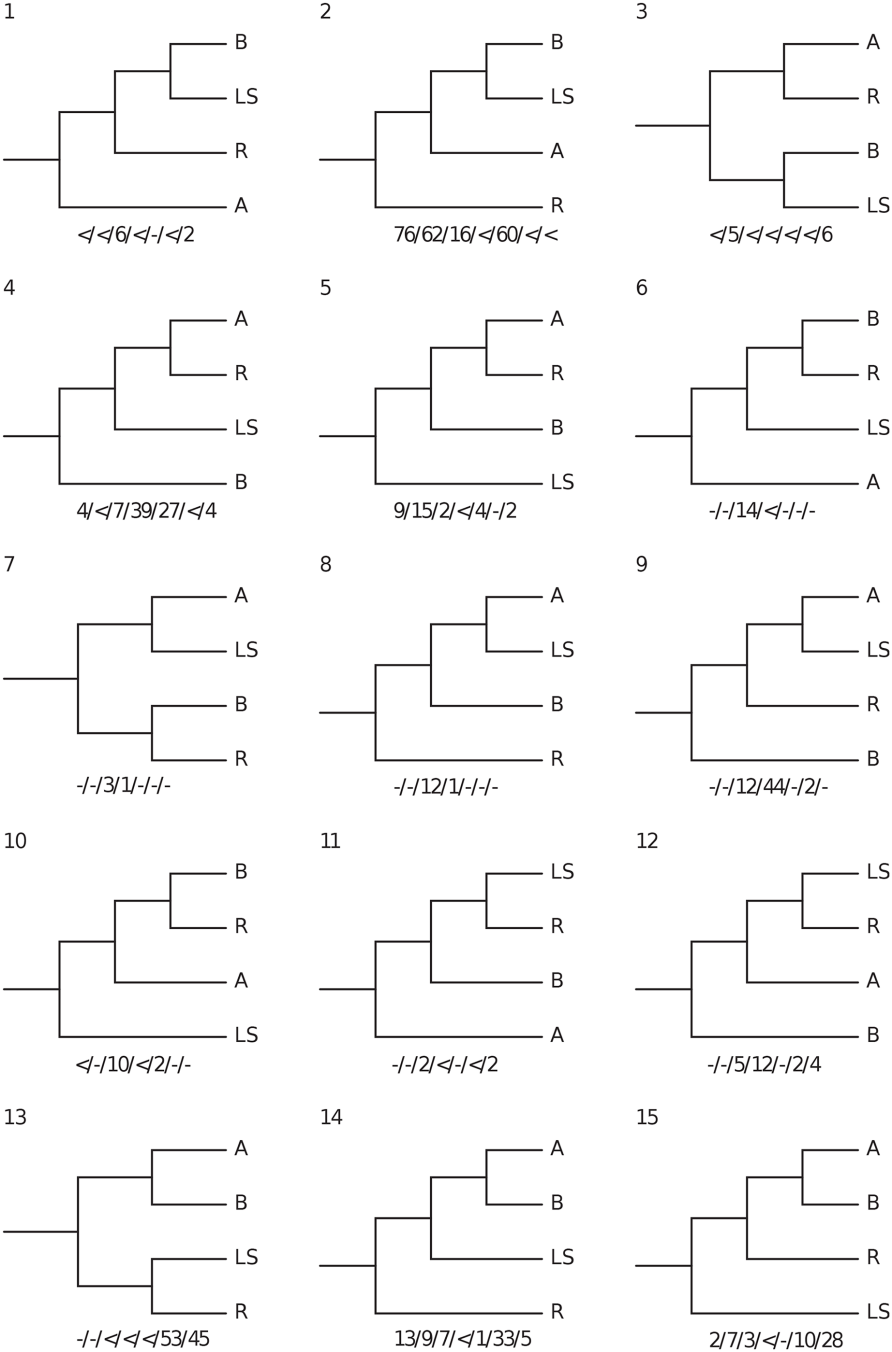
The fifteen rooted trees for the four taxa used to assess taxon instability and their percentage frequency of occurrence in 1000 Bayesian or Bootstrap (LogDet) trees. Taxa abbreviated as follows: *Grandisonia alternans* (A), *Hypogeophis brevis* (B) *Grandisonia larvata* + *Grandisonia sechellensis* (LS) and *Hypogeophis rostratus* (R). Numbers below trees are support values for analyses of: all nucleotides / protein-coding nucleotides / tRNAs / rRNAs /amino acids / LogDet for all data / LogDet for protein-coding data. < = less than 1% support,— = zero support.

We found no evidence of sequence saturation, but both the protein-coding and the full nucleotide datasets showed significant base compositional heterogeneities (not shown) and were thus analysed also with LogDet distances. For each dataset and partitioning strategy, the BI and ML analyses recovered the same set of phylogenetic relationships (Fig 1). All analyses agreed in providing maximal support for the monophyly of each species that was represented by more than one individual (i.e., all Seychelles species except *Hypogeophis brevis*) and for a sister group relationship between *Grandisonia larvata* and *G*. *sechellensis*, but otherwise relationships among the species were resolved variably in the different analyses and generally with only low support. Accepting the rooting of the Seychelles caecilian tree with *Praslinia cooperi* and collapsing *G*. *larvata* + *G*. *sechellensis* into a single taxon reduces the remaining interrelationships to a four-taxon problem, for which there are 10 possible clades and 15 distinct rooted trees. Table 6 summarises the support for these 10 clades across different analyses. All 10 possible clades occur across the bootstrap/Bayesian trees but several clades are never supported by more than 50% of the trees from any single analysis. Using the notation A = *G*. *alternans*; B = *H*. *brevis*; L = *G*. *larvata* + *G*. *sechellensis*; R = *H*. *rostratus*, the groupings that never receive majority support are AL, AR, ARL, ABR, BR, and BLR (Fig 2). Two hypotheses, AB and LR, have majority support only in LogDet analyses, highlighting the potential for the moderate to high support for some conflicting hypotheses (e.g. ALR and BL) to be an artefact of base compositional biases in these data. Unsurprisingly, analyses of the smallest dataset (tRNA) yield the smallest maximum support values for any clade. Fig 2 provides a complementary summary of the frequency of occurrence of all possible 15 rooted trees. Note that only two of the 15 trees (trees 2 and 13) ever form a majority in any of the bootstrap analyses. Overall, the pattern of low to moderate support (that is not sustained across multiple analyses) suggests that the data are simply not sufficient for resolving relationships among these four taxa.

**Table 6:** Summary of percentage of support for clades presented in Fig 2. A = *G*. *alternans*, B = *H*. *brevis*, L = *G*. *larvata* + *G*. *sechellensis*, R = *H*. *rostratus*.—indicates zero support. Abbreviations in column 1 are as follows: BI = Bayesian Inference analysis; LD = LogDet analysis; All = complete nucleotide dataset; rRNA = rRNA dataset; tRNA = tRNA dataset; PC = protein coding nucleotide dataset; AA = amino acid dataset.

Analysis	AB	AL	AR	ABL	ABR	ALR	BL	BR	BLR	LR
BI All	15.1	-	8.2	88.9	6.8	3.9	76.4	0.6	0.1	-
LD All	95.3	0.2	1.2	33.4	9.8	3	1.5	-	0.5	55.1
BI rRNA	1.2	46.1	39.5	1.9	1.1	94.3	0.7	2.4	0.8	12,0
BI tRNA	10.2	27.6	9.6	35.6	14.2	24	22.9	26.3	22.2	7.4
BI PC	16.2	-	21	71.2	22.4	0.8	67.9	-	0.5	-
BI AA	1	-	36.4	61.2	11.5	26.7	60.8	2.4	-	-
LD PC	78.4	-	12.1	5.4	30.2	8.5	8.7	-	4.6	52.1

Comparisons of support for full and partial splits across the various analyses (Table 6) provide no indication that instability associated with any specific 'rogue’ taxon is obfuscating support for otherwise well-supported partial splits.

## Discussion

### NGS mitogenomics

In our experience, the overall most cost-effective method for obtaining mitochondrial genomes when total time and accuracy were taken into account was the shotgun sequencing approach with the Illumina MiSeq platform (Table 7). Although sequencing costs are much lower for generating complete mitogenomes with long-amplicon, multiplex and Sanger sequencing, it is more time intensive in terms of bench work and sequence handling. The multiplex data provide a much more enriched sample set but they require a large amount of time and, particularly for the Illumina HiSeq data, more computing power to process the data. Our multiplex data were not individually indexed, which increased the time required to reconstruct mitogenomes, and made it impossible to ensure with absolute certainty that all the constituent fragments in each reconstructed mitogenome pertain to a single individual specimen. In our case, we were able to partly address the latter concern because our multiplex datasets included only one sample of each species and because mitogenome sequences of the same specimen and/or conspecifics or close relatives were available as references. The performance of the long-amplicon approach could be improved by individually indexing samples, and although more accurate mitogenomic reconstructions could be accomplished, it must be noted that this would be with increased cost. Although the Illumina MiSeq is probably the most expensive method that we used per sample (~$430, in a total sample of six), it is fast for generating mitogenomes in terms of time required for lab work, sequencing and post-sequencing analysis and reconstruction. However, some MiSeq samples lacked high sequence coverage (<10x) when compared with multiplex sequencing on the Illumina HiSeq, so we would not recommend sequencing additional samples in organisms with similar genome sizes to reduce costs. In addition, and because the samples were indexed, our MiSeq approach allowed us to attribute sequenced fragments to the mitogenome of each individual with almost complete certainty (assuming lack of contamination). This shotgun sequencing method also provides data that can be used for other purposes, such as development of anonymous nuclear loci [25], future development of microsatellite markers [50] or for SNP identification [51].

**Table 7:** Comparison of performance of five approaches for generating our mitogenome sequence data from eight samples of Seychelles caecilians. Approximate relative ‘values’ depicted are * = low, ** = moderate, *** = high.

Method	Sequencing	Sample preparation time	Sample preparation cost	Sequencing running time	Sequencing cost	Mitogenome reconstruction time	Total time expenditure
Traditional	Sanger	***	**	*	*	***	***
Shotgun	Illumina MiSeq	*	*	**	**	*	*
Multiplex	Illumina HiSeq	***	**	***	***	***	**
Multiplex	Roche 454	***	**	**	**	**	**
Multiplex	Ion Torrent	***	**	*	*	**	**

### Molecular phylogeny and systematics of Seychelles caecilians

Our analyses suggest that mitogenomic data alone are not sufficient for resolving all phylogenetic relationships among Seychelles caecilians. One potential problem is substantial base-composition heterogeneity in the protein-coding genes, something that can mislead phylogenetic inference [52]. That LogDet, which can overcome base-composition heterogeneity, produced substantially different results to other methods (Table 6) does not allow us to discount this possibility. It is noteworthy that the pairing of *Hypogeophis rostratus* and *H*. *brevis* is almost never supported, and this calls into question the taxonomy proposed by Wilkinson *et al*. [22]. However the inadequacy of the data seems to preclude ruling out anything at this stage other than relationships that contradict the well-supported sister-group relationship between *Grandisonia larvata* and *G*. *sechellensis* that was found in many previous analyses also [16,17,21,23,24]. With additional sampling (e.g., a second individual of *H*. *brevis*) there is the potential to improve the resolution of Seychelles caecilian phylogeny based on mitogenomes, but it seems more likely that the remaining phylogenetic problems will require additional sequence data from nuclear genes.
